# Cone Dystrophies: An Optical Coherence Tomography Angiography Study

**DOI:** 10.3390/jcm9051500

**Published:** 2020-05-16

**Authors:** Lisa Toto, Maurizio Battaglia Parodi, Rossella D’Aloisio, Stefano Mercuri, Alfonso Senatore, Luca Di Antonio, Guido Di Marzio, Marta Di Nicola, Rodolfo Mastropasqua

**Affiliations:** 1 Ophthalmology Clinic, Department of Medicine and Science of Ageing, University G. D’Annunzio Chieti-Pescara, 66100 Chieti, Italy; l.toto@unich.it (L.T.); doctor_alf@hotmail.it (A.S.); monsieurluca@yahoo.com (L.D.A.); dimarzio61@alice.it (G.D.M.); 2 Scientific Institute San Raffaele, University Vita-Salute, IRCCS, 20131 Milano, Italy; battagliaparodi.maurizio@hsr.it (M.B.P.); mercuristef@gmail.com (S.M.); 3 Department of Medical, Oral and Biotechnological Sciences, Laboratory of Biostatistics, University “G. d’Annunzio” Chieti-Pescara, 66100 Chieti, Italy; mdinicola@unich.it; 4 Institute of Ophthalmology, University of Modena and Reggio Emilia, 41121 Modena, Italy; rodolfo.mastropasqua@gmail.com

**Keywords:** cone dystrophies, optical coherence tomography angiography, retinal vessel density

## Abstract

Background: This study investigates the relationship between retinal vascularization and macular function in patients with cone dystrophies (CDs). Methods: Twenty CD patients (40 eyes) and 20 healthy controls (20 eyes) were enrolled in this prospective case-control study. Patients underwent full ophthalmological examination, microperimetry, full-field, pattern and multifocal electroretinogram (ERG, PERG, mfERG) and optical coherence tomography angiography (OCTA). Main outcome measures were as follows: foveal and parafoveal inner and outer retinal thickness; microperimetry sensitivity in the central 4° and 8°, ERG b wave amplitudes and peak times, PERG P50 and N95 amplitudes and latencies, and mfERG N1 to P1 amplitudes; and superficial capillary plexus (SCP), deep capillary plexus (DCP) and choriocapillary (CC) plexus vessel densities, divided into foveal and parafoveal region. Results: Retinal thickness, SCP and DCP densities were significantly related to PERG. A significant relationship was found between foveal and parafoveal retinal thicknesses and foveal SCP density (*p* < 0.001 and *p* = 0.018, respectively) and between parafoveal retinal thickness and parafoveal SCP density (*p* = 0.002). Foveal and parafoveal retinal thicknesses were significantly related to parafoveal DCP density (*p* = 0.007 and *p* < 0.001). Foveal and parafoveal retinal thicknesses, foveal SCP and parafoveal DPC densities were significantly reduced in CD patients compared to controls (*p* < 0.001; *p* = 0.010 and *p* = 0.008, respectively). PERG and mfERG amplitudes were significantly reduced in CD patients compared to controls (*p* < 0.01). Conclusions: CD eyes showed reduced retinal thickness significantly related to reduced vessel density, possibly caused by a decreased metabolic demand. In addition, vessel density significantly correlated with loss of function.

## 1. Introduction

Cone dystrophies (CDs) are a group of rare inherited pathologies that are characterised by cone dysfunction and loss. No explicit classification of CDs is available because some authors use this term to describe only progressive forms, while others use it to describe both stationary and progressive forms [1,2]. According to the involvement of macular cone receptors, patients typically present with photophobia, decreased central vision, dyschromatopsia and occasionally nystagmus, with typical progression to legal blindness by middle age. Autosomal-dominant, autosomal recessive and X-linked modes of inheritance have all been reported [2,3,4].

Full-field electroretinogram (ERG) is considered the gold standard for diagnosis of CDs, revealing marked depression of cone responses with normal rod function or possible late rod involvement in CDs [5]. Retinal phenotypic manifestations are variable. Fundus autofluorescence (FAF) may highlight a “bull’s-eye” ring of hyperautofluorescence or a geographic zone of hypoautofluorescence, associated with possible central thinning with diffuse central or paracentral ellipsoid zone (EZ) attenuation or loss at structural optical coherence tomography (OCT) [6,7]. 

OCT angiography (OCTA) is a new dyeless retinal imaging technique that assesses retinal and choroidal microvasculature. It has already been used to investigate vasculature alterations in the superficial capillary plexus (SCP), deep capillary plexus (DCP) and choriocapillaris (CC) in several retinal vascular diseases and hereditary retinal photoreceptor degeneration diseases, such as retinitis pigmentosa [8,9,10]. 

The aim of our study was to investigate the status of retinal and CC microvasculature and the possible relationship between retinal vascularization and function using OCTA, ERG, multifocal ERG (mfERG) and pattern ERG (PERG) in a cohort of patients with progressive forms of CDs.

## 2. Methods

### 2.1. Study Participants

Twenty patients with diagnoses of CD confirmed by genetic analysis, clinical features at fundus observation, electrophysiological evidence, FAF, and spectral-domain OCT (SD–OCT), followed by the Electrophysiology Service of the Ophthalmology Clinic, Department of Medicine and Science of Ageing, University “G. d’Annunzio” Chieti- Pescara, and by the Dystrophy Unit, Ophthalmology Department, Ospedale San Raffaele, Milan, were enrolled in this retrospective case series. Characterization of allelic variants was investigated by means of a targeted next-generation sequencing (NGS) panel.

Clinical diagnostic criteria for CD diagnosis were as follows: possible evidence of Mendelian inheritance; visual symptoms such as decreasing central visual acuity, photophobia, dyschromatopsia and nystagmus; reduced photopic responses at ERG flash; bull’s-eye maculopathy or macular retinal pigment epithelium (RPE) alterations at fundus examination; “bull’s-eye” ring of hyperautofluorescence or a geographic zone of hypoautofluorescence at FAF, central thinning with central or paracentral EZ attenuation or loss at OCT.

Exclusion criteria were as follows: (1) best-corrected visual acuity (BCVA) lower than 1.0 logarithmic minimum angle of resolution (logMAR); (2) any retinal dystrophy or retinal disease other than CDs; (3) any optic neuropathy, including glaucoma, or any condition increasing the risk of secondary glaucoma; (4) intraocular pressure (IOP) greater than 21 mmHg; (5) medium lens opacities (according to Lens Opacities Classification System).

The study was approved by the Institutional Review Board of both ophthalmologic centres and adhered to the tenets of the Declaration of Helsinki, with written informed consent signed for all patients willing to participate in the study (2019/CD01).

### 2.2. Procedures

Patients underwent full ophthalmologic examination, including BCVA evaluation, using Early Treatment Diabetic Retinopathy Study (ETDRS) charts, Goldmann applanation tonometry, slit-lamp biomicroscopy and indirect fundus ophthalmoscopy. In addition, all patients underwent full-field ERG, mfERG and PERG with the Retimax (CSO, Scandicci, Florence, Italy), microperimetry (MP) by means of an MP-1 Microperimeter (Nidek Technologies, Padova, Italy), SD OCT (Spectralis, HRA Heidelberg, Heidelberg, Germany) and OCTA (Angiovue, Optovue, Freemont CA, USA).

Color vision assessment was performed using the Ishihara pseudoisochromatic plates and the Farnsworth Munsell 100-hue test. 

### 2.3. Electrophysiology Testing

ERG, PERG and mfERG were recorded for each patient according to the International Society for Clinical Electrophysiology of Vision (ISCEV) protocols [11,12,13]. The amplitudes and peak times of b waves in dark-adapted 0.01 ERG, dark-adapted 3.0 ERG and light-adapted 3.0 ERG and the amplitude of the light-adapted 30 Hz flicker ERG were measured for all patients.

P50 and N95 amplitudes and latencies on PERGs were analysed for each patient. Furthermore, we analysed the ratio between N95 and P50 amplitudes (N95/P50). In the mfERG, the ocular fundus was segmented by an array of 61 hexagons testing a central retinal area of 25°, and average responses for the implicit times and amplitudes of N1 (first negative component) and P1 (first positive component) of the first-order kernel were calculated for five regional ring groups (R1 to R5). Amplitude was measured from the baseline to the trough (N1) or the peak (P1) of the deflection. N1 to P1 amplitudes (from the first negative to the first positive peak) on mfERGs in the five recorded rings were considered for analysis. Patients were instructed to look at a fixation point incorporated into the stimulus dome for ERG or to the centre of a cross on a video display for PERGs and mfERGs to ensure accurate execution of the exam. Correct fixation was monitored with the help of an infrared CCD camera positioned in the ganzfeld stimulator during ERG or by direct observation of the patient fixating on the video display during PERG or mfERG acquisition.

### 2.4. Microperimetry

Microperimetry was performed by means of the MP-1 microperimeter. All patients were dilated with tropicamide 1% eye drops, and after pre-test training, 5 minutes were allotted for adaptation to the dark. This test is routinely carried out with an automated eye-tracking system, which provides real-time compensation for eye movements and allows improved presentation of a stimulus at a predefined retinal location. During the test, the patient was encouraged to fixate on a red cross target, 2° in diameter, on a white monochromatic background at 4 asb. Then, retinal sensitivity was tested using a customised radial grid centred on the fovea with 77 Goldman III stimuli covering the central 20°. The stimulus intensity ranged from 0 dB to 20 dB (0 dB corresponded to the strongest signal intensity of 127 cd/m^2^) in 1-dB steps, and the duration of each stimulus was 200 ms. To assess central macular retinal sensitivity, differential light threshold values were compared by calculating the mean of the central 4° and 8° of the macular area, which was averaged automatically by the MP-1 software programme for the mean sensitivity in a polygon. To assess fixation, fundus movements were tracked during the examination, and where the patient gazed on the fixation target and the fixation pattern were assessed. To evaluate fixation location, the fixation target centred on the fovea was defined. Fixation location was expressed as a percentage of fixation points located within the 2° and 4° central areas. Eyes with >50% of the preferred fixation points located within the central area were classified as having predominantly central fixation. Eyes with >25% but <50% of the preferred fixation points located within the central area were classified as having poor central fixation. Eyes with <25% of the preferred fixation points located within the central area were classified as having predominantly eccentric fixation.

### 2.5. Optical Coherence Tomography Angiography

Microvascular retinal and choroidal characteristics of CD patients were evaluated with RTVue XR Avanti SD-OCT device with AngioVue software (version 2018.1.0.24; Optovue, Inc., Freemont, CA, USA).

Vascular retinal layers were visualised and segmented as previously described in the SCP, DCP and CC [8].

The projection-resolved algorithm was used to remove projection artifacts from the inner vascular plexus in the deep vascular plexus. This algorithm retains flow signals from blood vessels while suppressing projected flow signals in deeper layers. Images were reviewed by two investigators (LT and MBP) for segmentation accuracy; if segmentation errors were observed, then they were corrected using the segmentation and propagation tool from AngioVue (Angiovue, Optovue, Freemont CA, USA). Final images were reviewed again to confirm segmentation placement in all B-Scans.

Objective quantification of vessel density was carried out for each eye using SSADA software (version 2018.1.0.24; Optovue, Inc., Freemont, CA, USA). A quantitative analysis was performed on the OCTA en-face images for each eye using AngioVue software as previously described [8]. 

Vessel densities of the SCP, DCP and CC were evaluated in the foveal and parafoveal areas. Vessel density was defined as the percentage of the area occupied by vessels in a circular region of interest (ROI) of 3 mm in diameter positioned on the centre of the foveal avascular zone and including the foveal area (1 mm of diameter) and the parafoveal area, which constitute the remaining part inside the ROI.

Two independent observers subjectively examined vessel anomalies inside the 3 × 3 mm ROI on OCTA and evaluated vessel caliber (normal, narrowed or ectatic), the vessel course (normal or distorted) and density (normal, increased or rarified) of the superficial and deep retinal capillaries.

### 2.6. Foveal and Parafoveal Retinal Thickness Analysis

Foveal macular thickness (MT), parafoveal MT, parafoveal MT in the superior and inferior hemi-macular areas (S-Hemi MT and I-Hemi MT) from the ILM to the retinal pigment epithelium (ILM-RPE) (whole retina), parafoveal MT in the S-Hemi and I-Hemi areas from the ILM to the IPL (inner retina or ganglion cell complex, GCC) and parafoveal MT in the S-Hemi and I-Hemi areas from the IPL to the RPE (outer retina) were automatically calculated by software on OCTA 3 × 3 mm volume scans. 

A circular ROI centred on the Foveal Avascular Zone (FAZ) with a diameter of 3.0 mm was used for retinal thickness analysis: the central foveal area (1 mm in diameter) and the parafoveal area constituted the remaining part inside the ROI (full parafoveal area or parafoveal area in the temporal, superior, nasal and inferior quadrants).

### 2.7. Subfoveal Choroidal Thickness Analysis

The acquisition protocol for SD–OCT (Spectralis, HRA Heidelberg, Heidelberg, Germany) included a horizontal and vertical B-scans centered on the fovea with enhanced depth imaging (EDI) mode in all patients.

Subfoveal choroidal thickness (SFCT), measured vertically from the outer border of the RPE to the inner border of the sclera, was measured using the inbuilt manual caliper on EDI–OCT scans.

### 2.8. Main Outcome Measures 

The main outcome measures were foveal and parafoveal retinal thicknesses in the inner (from the ILM to the IPL), outer (from the IPL to the RPE) and whole (from the ILM to the RPE) retina; SCP and DCP densities and CC density, which were tested in the whole retina and the foveal and parafoveal areas; FAZ area; dark-adapted 0.01 ERG, dark-adapted 3.0 ERG and light-adapted 3.0 ERG b wave amplitudes and latencies and light-adapted 30 Hz flicker ERG; PERG P50 and N95 amplitudes and latencies; the ratio between N95 amplitude and P50 amplitude (N95/P50); mfERG N1 to P1 amplitude in the five recorded rings; retinal sensitivity on microperimetry at 4°, 8° and 20°; and fixation location within the 2° and 4° central areas. Correlation between morphological and functional parameters were evaluated in patients with CD.

### 2.9. Statistical Analysis

Power analysis was performed for testing the regression between retinal vascularization and function parameters in patients with CDs on the basis of previously observed data or published results. The power of the study was 80% considering *n* = 20, an effect size of 0.4 and an alpha error rate of 0.05.

The quantitative variables were summarised as the mean and standard deviation (SD) or the median and interquartile range (IQR) according to their distributions. Qualitative variables were summarised as frequencies and percentages. Shapiro–Wilk’s test was performed to evaluate departures from normal distribution for each variable. 

The Mann–Whitney *U* test was performed to evaluate significant differences in functional and morphological parameters between the CD patients and controls.

The effects of vessel density and retinal/choroidal thickness on functional parameters were analysed using different linear mixed models using the eye as the unit of analysis and considering the effect of the paired eye as a random effect. A linear mixed model was used to regress morphological and functional parameters on the fixed-effect factors assuming an unstructured covariance matrix. False discovery rate correction (FDR) was applied to control the family-wise type I error rate, and a q-value less than 0.05 was considered statistically significant. The statistical analysis was performed using IBM^®^ SPSS Statistics v 20.0 software (SPSS, Inc., Chicago, IL, USA).

## 3. Results

### 3.1. Demographic and Clinical Data 

Twenty patients (40 eyes, 12 females and 8 males) with diagnoses of CD and 20 controls (20 eyes, 11 females, 9 males) were enrolled in this retrospective case series.

The mean age of patients with CD was 32.25 ± 14.13 years (range 13–62 years), while the mean age of controls was 32.85 ± 13.61 years (range 17–63 years) (*p* = 0.874). Thirteen out of 20 patients had a confirmed genetic analysis, whilst seven patients refused the examination for personal reasons (Table 1).

### 3.2. Visual Acuity, Electrophysiology and Microperimetry

The mean best-corrected visual acuity (BCVA) values were 0.7 ± 0.3 logMAR in the CDs group and 0.0 ± 0.1 logMAR in the control group (*p* < 0.001).

**Figure 1:**
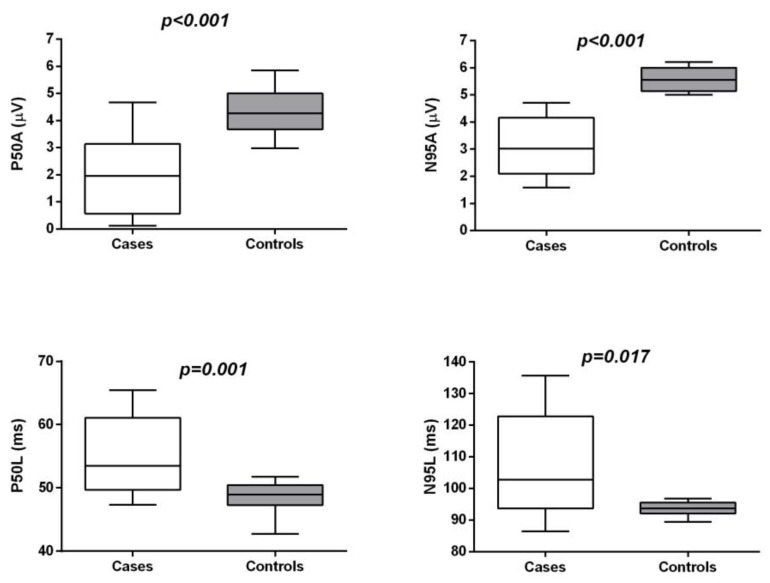
Box–whiskers graphs of functional parameters. Box–whiskers plots show the 25th and 75th percentiles (box) with 95% Tukey confidence intervals (whiskers) and median values (transverse lines in the box). *p*-values in the figure are derived from the Mann–Whitney *U* test.

PERG P50A and N95A were significantly reduced, and P50L and N95L were significantly delayed in CD patients compared to the controls (*p* < 0.01) (Table 2 and Figure 1).

MfERG N1P1A was significantly reduced in five regional rings in CD patients compared to the controls (*p* < 0.001) (Table 2).

Dark-adapted 0.01 ERG was not significantly different compared to the control, group, dark-adapted 3.0 ERG and light-adapted 3.0 ERG b wave amplitudes and latencies were significantly reduced, and light-adapted 30 Hz flicker ERG amplitude was also significantly reduced; on the contrary, there was not a significant difference of 30 Hz flicker ERG latency between cases and controls (Table 2). Three patients (6 eyes) with KCNV2 gene mutations showed supernormal dark-adapted 0.01 and 3.0 ERG amplitudes.

Retinal sensitivity on MP at 4° and 8° was significantly reduced in patients with CDs compared to that in the normal subjects (Table 2). Fixation location points inside the 2° and 4° central area were predominantly central in both groups.

### 3.3. OCT Angiography and SD–OCT Analysis

**Figure 2:**
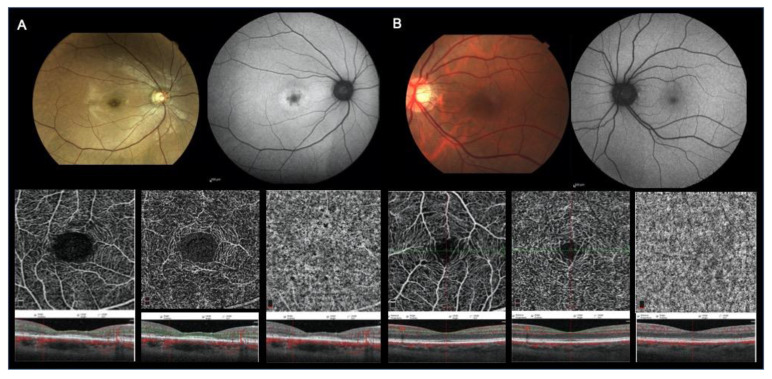
Multimodal imaging of a cone dystrophy eye of 42-year-old patient and a normal control eye of a 48-year-old patient (**A** and **B** images, respectively). (**A**) Colour fundus photograph (CFP) showing central retinal pigment epithelium (RPE) changes (first row, left); fundus autofluorescence (FAF) revealing central areas of increased autofluorescence (first row, right); optical coherence tomography angiography (OCTA) of the superficial capillary plexus (SCP) (second row, left), deep capillary plexus (DCP) (second row, middle) and choriocapillaris (CC) (second row, right) showing some interruptions of the perifoveal anastomotic arcade in the superficial plexus and no apparent modifications of the deep plexus and choriocapillaris; corresponding structural SD–OCT images from vertical and horizontal scans centred on the fovea (third row left, middle and right with overlying segmentation bands at the level of the SCP, DCP and CC, respectively) showing central subfoveal external limiting membrane and inner segment ellipsoid band defects, photoreceptor outer segment defects and slightly thin subfoveal RPE/Bruchs complex. (**B**) CFP (first row left), FAF (first row right), OCTA of the SCP, DCP and CC (second row left, middle and right respectively) and SD–OCT (third row left, middle and right with overlying segmentation bands at the level of the SCP, DCP and CC, respectively) of a normal eye.

Retinal microcirculation was visualised in detail in all cases. In all patients (40 eyes) in the CDs group, vessels were rarefied to various extents in the foveal and parafoveal areas in both SCP and DCP. The vessel calibre and course did not show any alterations. An increased foveal avascular area due to interruption of the perifoveal anastomotic arcades was evident in the SCP and DCP (Figure 2).

SCP density was mainly reduced in the foveal area (*p* = 0.010), and DCP density was mainly reduced in the parafoveal area (*p* = 0.008), compared to the control group. The CC density did not show significant differences between the two groups (Table 3).

The FAZ area was significantly larger in CD patients compared to that in the controls (Table 3).

Reductions in retinal thickness were observed in both the foveal and parafoveal areas in CD patients compared to that in the controls (*p* < 0.001); this reduction was observed in the whole, inner and outer retina (*p* < 0.001). 

The SFCT was 251.0 µm (203.0–269.2) in CDs and 272.0 µm (226.0–332.0) in controls (*p* = 0.101).

### 3.4. Regression Analysis of Different Parameters

PERG (N95) was significantly related to parafoveal retinal thickness (whole retina and inner retina) and to parafoveal SCP and DCP densities (Table 4 and Table 5).

Parafoveal retinal thickness and parafoveal SCP and DCP densities showed a trend toward a significant prediction of mfERG (N1P1A ring 5) (Table 4 and Table 5).

A direct relationship was found between whole-retina thickness in the foveal and parafoveal areas and foveal SCP density (b = 4.30; *p* < 0.001 and b = 2.33; *p* = 0.018, respectively) as well as between whole-retina thickness in the parafoveal area and parafoveal SCP density (b = 4.25; *p* = 0.002) (Table 6). 

A significant relationship was also found between inner retinal thickness in the parafoveal area and parafoveal SCP density (b = 2.59; *p* = 0.002) (Table 6). A significant relationship was found between whole-retina thickness in the foveal and parafoveal areas and parafoveal DCP density (b = 4.93; *p* < 0.001 and b = 4.99; *p* < 0.001, respectively) as well as between inner retinal thickness in the parafoveal area and parafoveal DCP density (b = 3.41; *p* = 0.005) (Table 6). There was no significant correlation between SFCT and BCVA (b = –0.23; *p* = 0.621).

## 4. Discussion

In this study we investigated the retinal and CC microvasculature status in patients with CDs using OCTA and evaluated the correlation between anatomical and functional data by means of electrophysiology testing. Our group of eyes with CDs was characterised by a reduced retinal vessel density, which was related to reduced retinal thickness. Moreover, we demonstrated a direct correlation between reduced vessel density and loss of function, as documented by PERG and mfERG impairment. 

Retinal and choroidal vessel alterations have been observed in rod–cone dystrophy using OCTA, characterised by SCP, DCP and CC density reduction in the macular area and in the mid-peripheral retina when assessed using widefield OCTA. This evidence confirmed previous clinical findings of vessel changes in retinitis pigmentosa characterised by vessel attenuation [14,15,16,17]. 

Clinical evidence of vessel alterations in selective or prevalent cone loss, such as CDs or cone–rod dystrophy (CRD), is very limited in the literature. Vessel attenuation has been reported in patients with CRD on fundus examination [18]. 

In our series, we observed reductions in vessel density in the SCP, mainly in the foveal area, and in the DCP in the parafoveal area of CDs patients, whilst alterations of choriocapillaris vessel density were not found.

In addition, a reduction in both inner retina (GCC complex) and outer retina thickness were observed in the CD patients compared to controls. The vessel density alterations, particularly those in the DCP, significantly correlated with macular thickness. The macular function was significantly related to SCP and DCP densities and to whole-retinal and inner-retinal thickness. PERGs were significantly related to parafoveal DCP density, and MfERG showed a trend toward a significant relationship with parafoveal retinal thickness and parafoveal SCP and DCP densities. 

It has been previously reported that in CDs, reduced retinal thickness is mainly related to thinning of the outer nuclear layer [19,20].

In our study, both inner retina (GCC complex) and outer retina were reduced. 

Several authors have investigated the relationship between GCC, RNFL and bipolar cell dysfunction and photoreceptor loss [21,22,23,24].

Reductions in inner retinal layers due to inner retinal cell apoptosis, such as ganglion cell apoptosis after photoreceptor degeneration, have been described in inherited retinal diseases (IRDs), such as RP [23,24].

In addition, in prominent cone dysfunction diseases, such as cone–rod dystrophy, transsynaptic degeneration with involvement of the inner retinal layers as a consequence of outer retinal photoreceptor cell degeneration has been suggested [25,26]. A hypothesis of vessel remodelling after RGC loss in a retinal model of retinal degeneration has been postulated [27].

Differently from disorganisation of the retinal inner layers (DRIL) observed in retinal pathologies such as diabetic retinal edema and retinal vein occlusion retinal edema where boundaries between the ganglion cell–inner plexiform layer complex, inner nuclear layer, and outer plexiform layer cannot be identified for a certain extent, in CDs, the inner and outer retina show reduced thickness with preserved retinal interfaces in the inner part and absence of outer retinal structures to a different extent such as ELM and EZ band, or reduced thickness of photoreceptor outer segment and RPE [28].

Macular vessel depletion of CDs is also typical of other hereditary macular dystrophies such as Stargardt disease (STDG) [29,30]. Patients with advanced STGD showed a reduction of SCP, DCP and CC compared to healthy eyes related to a reduction of inner and outer retinal thickness.

We hypothesise that vessel depletion of the SCP and DCP of CD patients is a secondary event of inner retinal and outer retinal atrophy due to a reduced metabolic demand. Photoreceptors in healthy eyes have a high oxygen consumption, so progressive destruction of the outer segment of the retina in retinal dystrophies may reduce the demand for oxygen. Photoreceptor metabolism mainly depends on glycolysis and accounts for the majority of oxygen consumption in the retina, with cones containing 2-fold and 10-fold more mitochondria than rods in mice and primates, respectively, suggesting higher oxidative metabolism than rod [31,32]. The atrophy of the inner retina, probably due to the transsynaptic degeneration of RGC, additionally contributes to the depletion of inner retinal vascularization.

Choroid thinning has been described in IRDs, such as RP, rod–cone dystrophies and CDs, using SD–OCT, which is probably secondary to a loss of photoreceptor and RPE cells. Vascular endothelial growth factor, which is produced by RPE cells, has been demonstrated to participate in choroidal maintenance; therefore, it has been suggested to play a role in choroidal atrophy [33,34,35]. In addition, a reduction of the choriocapillaris density has been described in patients affected by RP using OCTA [10,17].

The normal subfoveal choroidal thickness and CC density detected in our study could be related to the stage of the disease or to the genotype of the included patients.

It is known that PERG P50 is partly ganglion cell-derived, with a contribution from structures distal to the GC in the visual pathways. The PERG N95 response is related to ganglion cell activity. In this study, the correlation between PERGs and parafoveal DCP density demonstrated that macular ischemia is related to poor macular function.

The alteration of PERG N95 observed in this study and the significant correlation between GCC thickness and N95 amplitude and latency confirm the occurrence of ganglion cell disfunction in CDs.

The MfERG response mainly derives from both bipolar and photoreceptor cells; however, a contribution from ganglion cells has also been hypothesised [36]. The alteration of MfERG and the relationship of this functional parameter with retinal thickness and SCP and DCP densities could be a confirmation of ganglion cell disfunction and of the possible role of macular vascular depletion on macular function. 

Our study has some limitations. One of the limits is the small sample size, although CDs are relatively rare diseases. In addition, possible segmentation failures that are characteristic of eyes with CDs could partially have reduced the reliability of vessel density measurements.

## 5. Conclusions

In conclusion, our data showed that our group of CD eyes was characterised by a reduced vessel density (mainly in the parafoveal area), which was related to reduced retinal thickness in both the inner and outer retina. Reduced retinal thickness is related to photoreceptor apoptosis and transsynaptic degeneration with involvement of the inner retinal layers as a consequence of outer retinal photoreceptor cell degeneration. The reduced vessel density could be a consequence of retinal atrophy due to a reduced metabolic demand. In addition, a direct correlation was found between vessel density and loss of function. These findings support the concept of vessel involvement as an important feature in the pathogenesis of CDs. The role of capillary loss in macular function deficits requires further investigation. 

Thus, OCTA could be used as an adjunctive tool to the armamentarium of the retinal specialists to monitor the progression of vascular involvement in cone dystrophy and correlate to the severity of vascular damage to the loss of function. CDs could be characterised by different severity of vascular depletion detectable using OCTA and could be classified in different phenotypes correlating with function. 

## Figures and Tables

**Table 1:** Age, gender and genetic profile of patients with cone dystrophies.

Patient ID	Gender	Age	Gene	Allele status	cDNA
1	F	44	Refused genetic testing		
2	M	62	ABCA4	homozygous	c.4297G > A
3	F	42	Refused genetic testing		
4	F	59	PROM1	homozygous	c.1354G > A
			RPGRIP1	homozygous	c.3821A > G
5	F	42	ABCA4	homozygous	c.1268A > C
6	M	13	ABCA4	heterozygous	c.5712A > T
			ABCA4	heterozygous	c.6449G > A
7	M	47	Refused genetic testing		
8	F	28	KCNV2	homozygous	c.1348T > A
9	M	29	ABCA4	homozygous	c.5882G > A
10	F	39	ABCA4	homozygous	c.1268A > C
11	M	22	PROM1	homozygous	c.1354G > A
			RPGRIP1	homozygous	c.3821A > G
12	M	20	PROM1	homozygous	c.1354G > A
			RPGRIP1	homozygous	c.3821A > G
13	M	27	KCNV2	homozygous	c.1348T > A
14	F	37	KCNV2	homozygous	c.1348T > A
15	F	20	ABCA4	homozygous	c.466A > G
16	F	15	ABCA4	heterozygous	c.5712A > T
				heterozygous	c.6449G > A
17	M	27	Refused genetic testing		
18	F	19	Refused genetic testing		
19	F	36	Refused genetic testing		
20	F	17	Refused genetic testing		

F: female; M: male; ABCA4: ATP-binding cassette, subfamily A, member 4; PROM 1: Prominin 1; RPGRIP1: retinitis pigmentosa GTPase regulator-interacting protein; KCNV2: potassium channel, voltage-gated, subfamily V, member 2.

**Table 2:** Functional parameters of cases and controls expressed as median and interquartile range.

Variable	Cases	Controls	*p*-Value ^a^
P50A (µV)	2.2 (0.7–3.1)	4.4 (3.6–5.1)	<0.001
P50L (ms)	53.4 (49.4–61.4)	49.0 (47.2–50.7)	0.001
N95A (µV)	3.2 (2.0–4.8)	56.1 (5.3–6.5)	<0.001
N95L (ms)	103.5 (93.4–123.4)	93.6 (92.1–95.8)	0.013
N95A/P50A	1.5 (1.0–4.4)	1.4 (1.3–1.7)	0.642
N1P1A Ring1 (µV)	93.1 (67.4–114.0)	143.1 (124.2–162.9)	<0.001
N1P1A Ring2 (µV)	39.1 (28.0–49.1)	63.4 (49.1–80.7)	<0.001
N1P1A Ring3 (µV)	17.4 (14.5–23.1)	31.3 (27.8–40.4)	<0.001
N1P1A Ring4 (µV)	12.1 (8.7–16.8)	23.2 (19.5–26.1)	<0.001
N1P1A Ring5 (µV)	8.3 (6.1–12.4)	17.1 (12.3–20.1)	<0.001
MP 4° (dB)	9.0 (3.4–13.8)	16.8 (15.0–18.5)	<0.001
MP 8° (dB)	12.0 (5.5–15.6)	14.2 (13.4–16.8)	0.004
MP 20° (dB)	13.5 (5.4–14.8)	11.0 (10.3–13.9)	0.933
Fixation 2° (%)	100.0 (93.0–100.0)	100.0 (100.0–100.0)	0.332
Fixation 4° (%)	100.0 (100.0–100.0)	100.0 (100.0–100.0)	0.999
dark–adapted 0.01 ERG A	153.7 (77.2–187.5)	200.5 (184.5–229.3)	0.004
dark–adapted 0.01 ERG L	79.3 (71.3–84.0)	74.3 (71.3–79.5)	0.501
dark–adapted 3.0 ERG A	143.0 (71.2–192.1)	207.2 (190.9–227.9)	0.004
dark–adapted 3.0 ERG L	44.0 (41.6–4.4)	40.8 (39.5–42.9)	0.039
light–adapted 3.0 ERG A	30.8 (11.0–88.8)	129.0 (112.0–139.3)	<0.001
light–adapted 3.0 ERG L	34.7 (33.8–35.8)	30.7 (29.3–33.0)	<0.001
**light–adapted 30 Hz flicker ERG A**	21.5 (13.2–35.5)	66.3 (59.5–83.8)	<0.001
**light–adapted 30 Hz flicker ERG L**	32.3 (30.5–36.7)	31.7 (29.3–34.0)	0.534

^a^ Mann–Whitney U test; bold value is significant after False discovery rate (FDR) correction.

**Table 3:** Morphological parameters of cases and controls expressed as median and interquartile range.

Variable	Cases	Controls	*p*-value ^a^
**Retinal thickness (ILM–RPE) µm**			
Fovea	162.0 (151.0–268.0)	251.0 (238.0–268.0)	<0.001
Parafovea	252.0 (224.0–327.0)	315.0 (298.5–325.3)	<0.001
S–Hemi	249.0 (224.5–285.0)	312.0 (300.5–330.0)	<0.001
I–Hemi	251.0 (228.0–281.0)	322.0 (300.0–331.0)	<0.001
**Retinal thickness (IPL–RPE) µm**			
Parafovea	154.0 (146.0–178.5)	196.5 (192.0–209.0)	<0.001
S–Hemi	157.5 (151.5–190.5)	196.0 (185.0–202.5)	<0.001
I–Hemi	157.0 (150.5–183.0)	197.3 (190.0–207.0)	<0.001
**Retinal thickness (ILM–IPL) µm**			
Parafovea	94.0 (77.0–111.0)	119.0 (114.0–125.0)	0.001
S–Hemi	93.5 (71.0–107.0)	118.5 (110.3–123.0)	0.001
I–Hemi	93.5 (83.0–111.0)	119.0 (113.0–125.0)	0.001
**Subfoveal choroidal thickness (SFCT) µm**	251.0 (203.0–332.0)	272.0 (226.0–332.0)	0.101
**Density of superficial capillary plexus %**			
Whole	50.0 (44.0–53.0)	50.4 (47.2–53.1)	0.523
Fovea	21.5 (18.0–38.5)	32.8 (27.3–38.5)	0.010
Parafovea	51.5 (45.0–55.0)	52.5 (48.5–54.8)	0.744
**Density of deep capillary plexus %**			
Whole	53.5 (47.5–62.0)	57.9 (56.3–61.5)	0.014
Fovea	32.0 (26.0–35.5)	28.3 (24.5–35.3)	0.550
Parafovea	55.3 (50.0–64.0)	61.3 (59.5–63.5)	0.008
**Density of choriocapillaris %**			
Whole	66.0 (65.0–68.5)	66.5 (65.3–67.8)	0.320
Fovea	68.5 (64.5–69.5)	66.3 (64.0–68.3)	0.301
Parafovea	66.0 (64.5–68.5)	65.3 (64.3–67.5)	0.633
**FAZ area (mm^2^)**	0.5 (0.4–0.8)	0.3 (0.2–0.4)	<0.001

^a^ Mann–Whitney U test; bold value is significant after FDR correction.

**Table 4:** Mixed-effects models using the eye as the unit of analysis performed to assess the effect of morphological parameters on functional parameters.

	ILM-RPE Thickness (Whole Retina)								ILM-IPL Thickness (Inner Retina)					
	Fovea		Parafovea		S-Hemi		I-Hemi		Parafovea		S-Hemi		I-Hemi	
	b (SE)	*p*-value	b (SE)	*p*-value	b (SE)	*p*-value	b (SE)	*p*-value	b (SE)	*p*-value	b (SE)	*p*-value	b (SE)	*p*-value
**N95A**	0.01 (0.01)	0.225	0.05 (0.01)	0.002 *	0.05 (0.01)	0.001 *	0.05 (0.01)	0.004 *	0.06 (0.03)	0.016 *	0.08 (0.03)	0.005 *	0.05 (0.03)	0.039
**N95L**	−0.09 (0.13)	0.459	−0.38 (0.12)	0.010 *	−0.35 (0.10)	0.011 *	−0.36 (0.11)	0.012 *	−0.61 (0.21)	0.025 *	−0.56 (0.20)	0.018 *	−0.59 (0.23)	0.032
**N1P1A Ring5**	−0.01 (0.04)	0.944	0.09 (0.05)	0.074	0.08 (0.03)	0.066	0.06 (0.06)	0.188	0.12 (0.07)	0.156	0.12 (0.05)	0.085	0.12 (0.09)	0.201

b: regression coefficient; SE: standard error; * significant after FDR correction.

**Table 5:** Mixed-effects models using the eye as the unit of analysis performed to assess the effect of morphological parameters on functional parameters.

	Density of Superficial Capillary Plexus				Density of Deep Capillary Plexus				Density of Choriocapillaris			
	Fovea		Parafovea		Fovea		Parafovea		Fovea		Parafovea	
	b (SE)	*p*−value	b (SE)	*p*−value	b (SE)	*p*−value	b (SE)	*p*−value	b (SE)	*p*−value	b (SE)	*p*−value
**N95A**	0.04 (0.05)	0.601	0.14 (0.08)	0.078	−0.04 (0.07)	0.742	0.18 (0.05)	0.015 *	−0.02 (0.16)	0.785	0.25 (0.14)	0.338
**N95L**	−0.42 (0.51)	0.398	−2.21 (0.57)	0.002 *	−0.57 (0.55)	0.287	−1.90 (0.50)	0.008 *	1.11 (1.40)	0.475	−0.52 (0.47)	0.733
**N1P1A Ring5**	0.05 (0.16)	0.861	0.42 (0.15)	0.028	0.10 (0.15)	0.578	0.44 (0.17)	0.039	−0.28 (0.41)	0.534	−0.58 (0.67)	0.639
**light−adapted 3.0 ERG A**	1.90 (1.18)	0.184	3.85 (1.40)	0.025	0.91 (1.15)	0.455	5.01 (0.89)	<0.001 *	−0.50 (2.67)	0.689	−1.29 (4.78)	0.801
**light−adapted 3.0 ERG L**	−0.11 (0.09)	0.421	−0.25 (0.14)	0.043	−0.09 (0.10)	0.547	−0.30 (0.11)	0.046	0.45 (0.22)	0.071	0.62 (0.33)	0.111
**light−adapted 30 Hz flicker ERG A**	−0.10 (0.51)	0.732	1.61 (0.54)	0.019	−0.03 (0.34)	0.899	1.31 (0.63)	0.099	−1.62 (1.17)	0.208	−1.66 (1.88)	0.532
**light−adapted 30 Hz flicker ERG L**	−0.16 (0.20)	0.423	−0.44 (0.13)	0.037	−0.06 (0.13)	0.758	−0.51 (0.14)	0.007*	−0.22 (0.41)	0.358	−1.90 (0.48)	0.067

b: regression coefficient; SE: standard error; * significant after FDR correction.

**Table 6:** Mixed-effects models using the eye as the unit of analysis performed to assess the effect of vascular density on retinal thickness.

	ILM-RPE thickness (whole retina)								ILM-IPL thickness (inner retina)					
	Fovea		Parafovea		S-Hemi		I-Hemi		Parafovea		S-Hemi		I-Hemi	
	b (SE)	*p*-value	b (SE)	*p*-value	b (SE)	*p*-value	b (SE)	*p*-value	b (SE)	*p*-value	b (SE)	*p*-value	b (SE)	*p*-value
**Density of superficial capillary plexus**														
Fovea	4.30(0.72)	<0.001 *	2.33(0.79)	0.018 *	2.30(0.90)	0.038	2.30(0.70)	0.016 *	0.92(0.55)	0.175	0.93(0.55)	0.108	0.86(0.48)	0.185
Parafovea	3.88(1.85)	0.073	4.33(0.99)	<0.001 *	4.25(1.20)	0.002 *	4.44(0.90)	<0.001 *	2.59(0.53)	0.002 *	2.58(0.58)	0.003 *	2.39(0.50)	0.002 *
**Density of Deep Capillary Plexus**														
Fovea	2.75(1.10)	0.036	1.44(0.90)	0.285	1.31(1.15)	0.481	1.50(0.95)	0.141	0.51(0.55)	0.432	0.49(0.63)	0.521	0.45(0.53)	0.501
Parafovea	4.98(1.55)	0.007 *	4.93 (0.59)	<0.001 *	4.99(0.69)	<0.001 *	4.91 (0.50)	<0.001 *	3.41(0.51)	0.005 *	2.51(0.48)	<0.001 *	2.36(0.52)	0.004 *
**Density of Choriocapillaris**														
Fovea	−2.09(3.65)	0.478	−0.87(2.63)	0.785	−1.20(2.87)	0.782	−0.70(2.55)	0.768	−1.33(1.48)	0.501	−1.34(1.59)	0.458	−1.27(1.44)	0.411
Parafovea	3.88(6.51)	0.589	1.78(4.98)	0.689	1.39(4.78)	0.598	2.38(4.60)	0.475	0.29(2.77)	0.958	0.37(2.95)	0.785	0.06(2.56)	0.902

b: regression coefficient; SE: standard error; * significant after FDR correction.

## Abbreviations

P50A	P50 amplitude
P50L	P50 latency
N95A	N95 amplitude
N95L	N95 latency
N1P1A	N1P1 amplitude
MP	microperimetry
ERG A	electroretinogram amplitude
ERG L	electroretinogram latency
ILM	internal limiting membrane
RPE	retinal pigment epithelium
IPL	internal plexiform layer
FAZ	foveal avascular zone
